# Complete Atrioventricular Block Presenting With Syncope Caused by Severe Hypothyroidism

**DOI:** 10.4021/cr221w

**Published:** 2012-09-20

**Authors:** Sang-Hoon Seol, Doo-Il Kim, Bo-Min Park, Dong-Kie Kim, Pil-Sang Song, Ki-Hun Kim, Han-Young Jin, Jeong-Sook Seo, Jae-Sik Jang, Tae-Hyun Yang, Dae-Kyeong Kim, Dong-Soo Kim

**Affiliations:** a Division of Cardiology, Department of Internal Medicine, Inje University College of Medicine, Haeundae Paik Hospital, Busan, Korea; b Division of Cardiology, Department of Internal Medicine, Inje University College of Medicine, Busan Paik Hospital, Busan, Korea

**Keywords:** Hypothyroidism, Syncope, Atrioventricular block

## Abstract

A 75-year-old man was admitted to our hospital with syncope. Electrocardiogram showed complete atrioventricular block and bradycardia with the minimum heart rate of 22 beats/ min. There was a possible indication for temporary cardiac pacemaker implantation. Laboratory data on admission revealed high TSH level with low free T4 level. To rule out functional atrioventricular block, we treated several days with thyroxine. A follow-up electrocardiogram showed improved heart rate without any atrioventricular block. We found that severe hypothyroidism caused a complete atrioventricular block with syncope, and thyroxine replacement completely improved these conditions.

## Introduction

Hypothyroidism is well known to be related to cardiac diseases [1]. Electrographic abnormalities associated with hypothyroidism include sinus bradycardia, flattened P waves, flat or inverted T waves, low voltage and delayed intraventricular conduction [2]. Symptomatic complete AV block requires a permanent pacemaker. Sometimes hypothyroidism causes complete atrioventricular (AV) block. We report a case of severe hypothyroidism with reversible AV block with presenting syncope.

## Case Report

**Figure 1:**
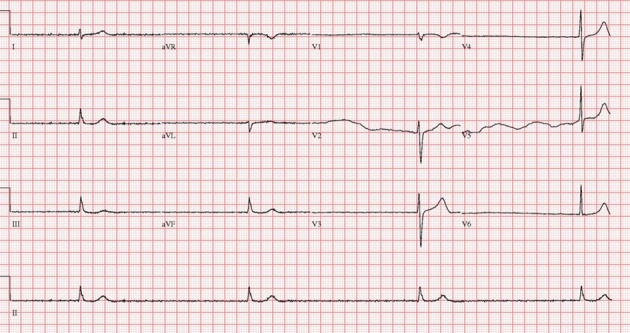
Electrocardiogram on admission revealed a third degree atrioventricular block with severe bradycardia.

**Figure 2:**
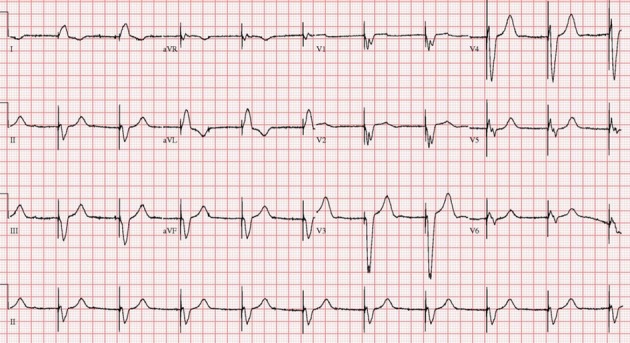
Electrocardiograms after temporary pacemaker implanted showed electronic rhythm.

**Figure 3:**
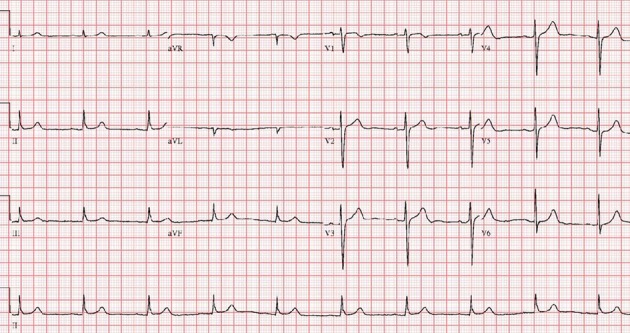
On 4 weeks electrocardiogram, complete atrioventricular block had completely disappeared.

A 75-year-old man was a smoker with no history of coronary artery disease, hypertension and hyperlipidemia except diabetes, which was diagnosed 3 years ago. He presented in the emergency room with syncope. The patient was conscious and physical examination showed heart rate 22 beats/min, blood pressure 110/70 mmHg, temperature 36.5 °C. Heart sound was normal without murmur and bruits. Initial electrocardiogram (ECG) showed complete AV block and bradycardia with minimum heart rate of 22 beats/min (Fig. 1). A chest X-ray was normal with mild pulmonary edema. Laboratory findings revealed severe hypothyroidism with a thyroid-stimulating hormone (TSH) level > 100 mIU/L (normal range 0.27 - 4.2) and a free T4 of 0.16 ng/dL (normal range 0.93 - 1.7). Hemoglobin (10.7 g/L) was mildly reduced. Kidney and liver enzymes were elevated (AST 120 IU/L, ALT 46 IU/L, LDH 464 IU/L, BUN 31.8 mg/dL, Creatinine 3.3 mg/dL). Transthoracic echocardiography showed normal left ventricular systolic function and mild tricuspid regurgitation. A temporary pacemaker was inserted via femoral vein with a ventricular rate of 60 beats/min (Fig. 2). Coronary angiography revealed no significant lesion. He was treated with low-dose levothyroxine. In 4 days AV conduction was recovery and the patient had a sinus rhythm of 48 beats/min. Kidney and liver function had normalized. He was discharged on the seventh day of admission. In four week, ECG revealed normal sinus rhythm with full recovery (Fig. 3). The TSH level had decreased to and the free T4 level was normal.

## Discussion

Hypothyroidism is characterized by a decrease in oxygen and substrate utilization by all the major organ systems of body. The cardiovascular changes of hypothyroidism are decrease in cardiac output and cardiac contractility, a reduction in heart rate, accelerated atherosclerosis, and an increase in vascular resistance [1]. Symptoms of cardiovascular dysfunction are uncommon in patients with hypothyroidism. Usual symptoms may include exertional dyspnea, cold intolerance, and fatigue. Findings on physical examination may include bradycardia, hypertension (diastole), nonpitting edema, and pleural or pericardial effusion [3]. Hypothyroidism may be a cause of complete AV block and ventricular tachycardia [4]. The mechanism of conduction disturbance in the heart remains unknown. Histopathologic finding of myocardium in myxedema heart is varied. There may be interstitial edema, myocardial fibrosis and mucinous vacuolization [5, 6]. The rapid normalization of electrocardiographic abnormalities after start of thyroxine implies reversible state. Among the various causes of AV block, hypothyroidism is one of the rare problems which can recover pharmacologically. There are some case reports such as our case [7-10]. However, failure to respond to thyroid hormone treatment is associated with irreversible extensive fibrotic change in myocardium [2]. In elderly patients, the prevalence of hypothyroidism varies from 1 to 17%, and, like many autoimmune disorders, is more common in [11]. The clinical presentation of hypothyroidism in elderly patients may be insidious. Therefore, hypothyroidism is frequently underdiagnosed in elderly patients. It is recommended that all patients with AV block of unknown origin receive a careful evaluation of thyroid function, especially in elderly patients before inserting a permanent pacemaker. We should replace thyroxine if hypothyroidism is diagnosed. We report severe hypothyroidism with high degree AV block, in which recovery to normal sinus rhythm following thyroid hormone replacement.
